# Recovery agenda for sustainable development post COVID-19 at the country level: developing a fuzzy action priority surface

**DOI:** 10.1007/s10668-021-01372-6

**Published:** 2021-04-03

**Authors:** Meisam Ranjbari, Zahra Shams Esfandabadi, Simone Domenico Scagnelli, Peer-Olaf Siebers, Francesco Quatraro

**Affiliations:** 1 Department of Economics and Statistics “Cognetti de Martiis”, University of Turin, Lungo Dora Siena 100 A, 10153 Turin, Italy; 2 Department of Environment, Land and Infrastructure Engineering (DIATI), Politecnico di Torino, Corso Duca degli Abruzzi 24, 10129 Turin, Italy; 3 Energy Center Lab, Politecnico di Torino, Via Paolo Borsellino 38/16, 10138 Turin, Italy; 4 School of Business and Law, Edith Cowan University, 270 Joondalup Dr, Joondalup, 6027 Australia; 5 School of Computer Science, University of Nottingham, Jubilee Campus, Nottingham, NG8 1BB UK; 6 BRICK, Collegio Carlo Alberto, Piazza Arbarello 8, 10123 Turin, Italy

**Keywords:** Sustainable development goals, COVID-19, Best–worst method, Multi-criteria decision-making, Iran, Action priority

## Abstract

As a response to the urgent call for recovery actions against the COVID-19 crisis, this research aims to identify action priority areas post COVID-19 toward achieving the targets of the sustainable development goals (SDGs) within the 2030 Agenda for Sustainable Development launched by the United Nations (UN). This paper applies a mixed-method approach to map the post-COVID-19 SDGs targets on a fuzzy action priority surface at the country level in Iran, as a developing country, by taking the following four main steps: (1) using a modified Delphi method to make a list of the SDGs targets influenced by COVID-19; (2) using the best–worst method, as a multi-criteria decision-making tool, to weight the COVID-19 effects on the SDGs targets achievement; also (3) to weight the impact of the SDGs targets on the sustainable development implementation; and finally (4) designing a fuzzy inference system to calculate the action priority scores of the SDGs targets. As a result, reduction of poor people proportion by half (SDG 1.2), development-oriented policies for supporting creativity and job creation (SDG 8.3), end the pandemics and other epidemics (SDG 3.3), reduction of deaths and economic loss caused by disasters (SDG 11.5), and financial support for small-scale enterprises (SDG 9.3) were identified as the highest priorities for action, respectively, in the recovery agenda for sustainable development post COVID-19. The provided fuzzy action priority surface supports the UN’s SDGs achievement and implementing the 2030 Agenda for Sustainable Development in Iran. It also serves as a guideline to help the government, stakeholders, and policy-makers better analyze the long-term effects of the pandemic on the SDGs and their associated targets and mitigate its adverse economic, social, and environmental consequences.

**Graphical abstract:**

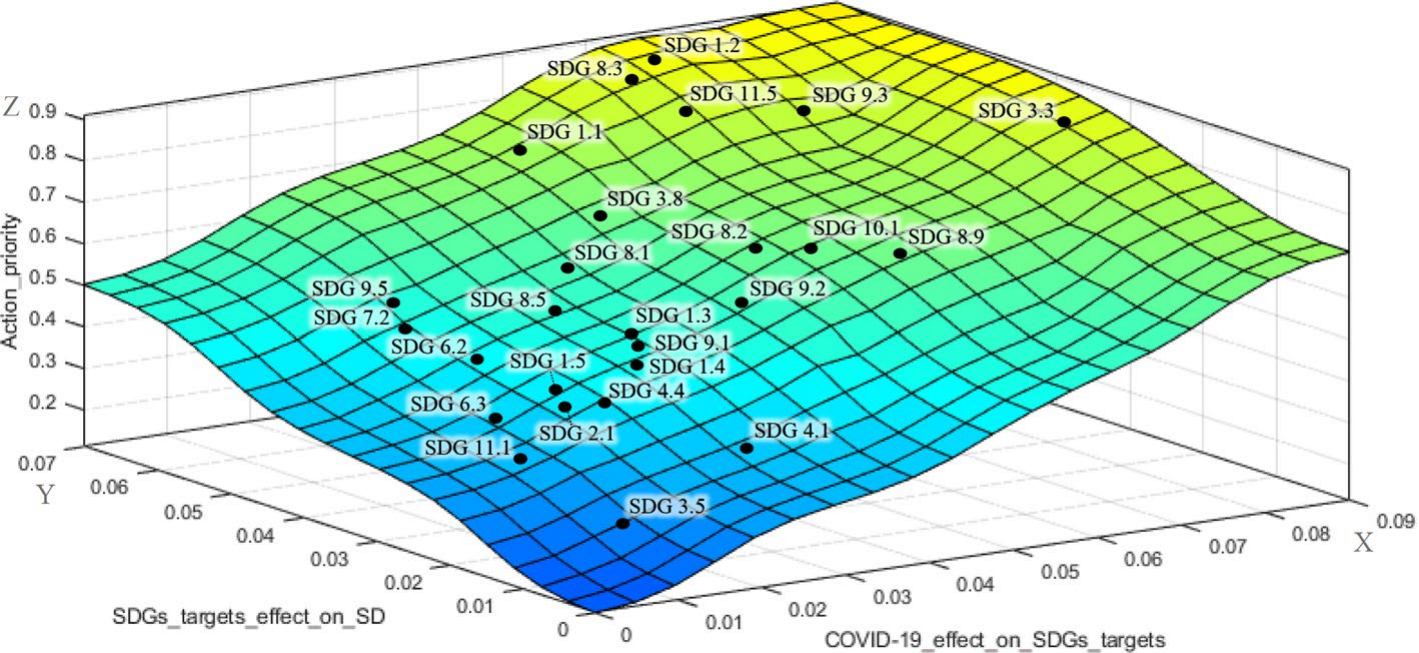

## Introduction

Since March 2020, the novel coronavirus-caused infectious disease (COVID-19) has become the most challenging topic to deal with for governments, industries, businesses, and people on a global scale. In March 2020, the World Health Organization (WHO) announced COVID-19 as the first pandemic caused by Coronavirus, which, as of March 14, 2021, has led to 119,220,681 confirmed cases and 2,642,826 deaths records in 235 countries, areas, or territories across the world (WHO, 2021a). This pandemic has been considered the most serious global health catastrophe of the century and the most challenging issue that the world is facing since the Second World War (Chakraborty & Maity, 2020). A wide range of business activities and the global economy are struggling with the COVID-19 restrictions and how to respond to the pandemic as fast as possible. During the COVID-19 crisis, most attention has been paid to the medical aspects of the pandemic, while the economic, social, and environmental consequences of the pandemic deserve to be investigated more in-depth (Gautam & Hens, 2020). However, the considerable implications of this pandemic have been increasingly studied by many scholars for a variety of different areas and disciplines such as transportation (Mogaji, 2020), renewable and sustainable energy (Hosseini, 2020), air pollution (Bherwani et al., 2020; Gautam, 2020; Gupta et al., 2020), health risk assessment (Ambade et al., 2021; Changotra et al., 2020; Gautam & Trivedi, 2020), tourism (Sigala, 2020), commodity markets (Rajput et al., 2020), and economic anxiety (Mann et al., 2020).

Such a crisis with global scope will also severely affect the achievement of the long-term global agreements between countries with shared action plans such as the 2030 Agenda for Sustainable Development launched by the United Nations (UN) in 2015 and the Paris agreement within the UN Framework Convention on Climate Change (UNFCC) signed in 2016. In September 2015, the UN General Assembly adopted the 2030 Agenda for Sustainable Development including 17 Sustainable Development Goals (SDGs) consisting of 169 targets on a variety of perspectives as a shared blueprint to address environmental, economic, and social dimensions of sustainable development (General Assemly, 2015). The SDGs offer a major opportunity for inclusive and transformative change (Siegel & Bastos, 2020) as well as a normative framework (Kumi et al., 2020) toward sustainability and creating dignity, peace, and prosperity for people and the planet (UN, 2018). The 2030 Agenda not only calls upon the governments but also industries and businesses from the private sectors to support the achievement of the SDGs (Van der Waal & Thijssens, 2020).

Sustainable development and the achievement of the SDGs and their associated targets are complex, broad, and integrated (Kumi et al., 2020) on the account of its interconnected goals and targets, which affect each other by nature (Ranjbari et al., 2019). The situation has become even more complex in light of economic pressure and difficulties emerged by the COVID-19 crisis. Therefore, the role of researchers for supporting governments and policy-makers in the global community has become more highlighted than before to respond to the urgent call for action as effectively as possible to this recent global shock.

Although limited research has been conducted within the sustainable development context considering the COVID-19 effects (Alibegovic et al., 2020; Barbier & Burgess, 2020; Bherwani et al., 2021; Yoshino et al., 2020), the lack of inclusive research on the effects of COVID-19 on the achievement of the targets of the UN SDGs at a country level is still a big issue. Moreover, the recovery strategies and planning for post-COVID-19 2030 Agenda for Sustainable Development for any country need to be in line with its resources and capacities, economic situation, and technological competencies and infrastructure. Iran is one of the highly impacted countries by COVID-19 in the world with 1,739,360 positive cases and 61,142 death records by March 14, 2021, reported to WHO (WHO, 2021b) and is experiencing a new wave of diagnosed cases starting from February 2021. According to the report provided by the World Bank Group (2020), the hardest-hit sectors by the COVID-19 pandemic in Iran are oil sales, travel, tourism, retails sales, manufacturing, and construction. Consequently, the achievement of the SDGs and accomplishment of the 2030 Agenda for Sustainable Development have been significantly challenged by the newly emerged situation. Therefore, an urgent priority action plan for Iran's government and sustainable development policy-makers within various domains is highly needed to support sustainable development blueprint after the pandemic.

This research aims at providing a post-COVID-19 recovery agenda toward sustainable development in Iran, as a developing country. In this regard, an action priority surface considering COVID-19 restrictions for achieving the SDGs targets is proposed by addressing the following four questions: (RQ1) Achieving which targets of the SDGs at the country level have been affected by COVID-19? (RQ2) How much is each one of those targets affected by COVID-19? (RQ3) How much does each one of those targets affect sustainable development? and finally (RQ4) Considering COVID-19 implications, what is the priority of action for each of the SDGs targets? The first question will be answered using a modified Delphi method, and the best–worst method (BWM) will be applied to respond to the second and third questions. Finally, for answering the fourth question, we will build a fuzzy inference system (FIS) and plot the action priority surface.

The remainder of the paper is arranged as follows: Section 2 briefly reviews the limited research on the SDGs and the COVID-19. Section 3 overviews the research design and explains the data analysis methods used. Section 4 presents the results of the analysis and discusses the main outputs and key findings. Finally, Sect. 5 concludes the research and delivers recommendations for future research on the COVID-19 implications for the 2030 Agenda for Sustainable Development.

## SDGs and COVID-19: an overview

Due to the recentness of the COVID-19 crisis, the research conducted on its impact on the achievement of the SDGs within the 2030 Agenda for Sustainable Development is still in its infancy. Only limited research has been carried out to study the effects of this current pandemic on the shared blueprint of SDGs focusing on the economic, social, and environmental pillars of sustainable development.

As an outcome of their research, Barbier and Burgess (2020) proposed three progress policies for developing countries toward several SDGs post COVID-19, including adopting subsidy swap for fossil fuel, implementing subsidy swap for irrigation to enhance sanitation and clean water, and a tropical carbon tax on fossil fuels to fund natural climate solutions. A qualitative analysis was conducted by Alibegovic et al. (2020) to investigate the impact of COVID-19 on the SDGs in Italy. They identified SDG 1 (no poverty), SDG 4 (quality education), and SDG 8 (decent work and economic growth) as the most impacted SDGs by COVID-19. In a theoretical study without any real data, Yoshino et al. (2020) highlighted the importance of government support and optimal portfolio allocation by institutional investors for the achievement of the SDGs in the post-COVID era. Moreover, Ranjbari et al. (2021) outlined the severe impacts of COVID-19 on the triple bottom line of sustainability and 2030 Agenda for Sustainable Development and highlighted the urgent need for actions to support SDGs achievement, particularly on the following directions: (1) sustainability transition opportunities in the wake of COVID-19 with a focus on SDG 12 and SDG 9, (2) innovative solutions for economic resilience to support SDG 1, SDG 8, and SDG 17, and (3) in-depth analysis of the COVID-19 long-term effects on social sustainability to achieve SDG 4, SDG 5, and SDG 10.

A statement made by the General Assembly (2015) postulates that many sectors in a country should work in harmony together to implement the 2030 Agenda for Sustainable development with a set of 17 wide-ranging SDGs and 169 targets from “No Poverty” to “Peace and Justice Strong Institutions.” Achieving the SDGs requires significant financial input (Ike et al., 2019) by governments and all other relevant stakeholders. Moreover, the recent financial burden imposed by COVID-19 on the global economy has made the issue more challenging, especially for developing countries. Therefore, effectively prioritizing the actions for sustainable development has become very crucial to overcome these newly emerged financial limitations. Consequently, the need for defining recovery strategies and identifying SDGs that should be prioritized for investment is currently at the highest level of importance for sustainable development policy-makers.

As the brief review of the literature indicates, there is an urgent need for a comprehensive study to prioritize the SDGs targets within the 2030 Agenda for Sustainable Development considering implications of COVID-19. In response to this need, our research, to our knowledge for the first time, investigates the COVID-19 implications for the achievement of the SDGs targets at the country level in Iran, as a developing country to prioritize them for action. The proposed action priority ranking considerably contributes to the recovery agenda for implementing sustainable development post COVID-19 in Iran.

## Research design and methodology

**Fig. 1:**
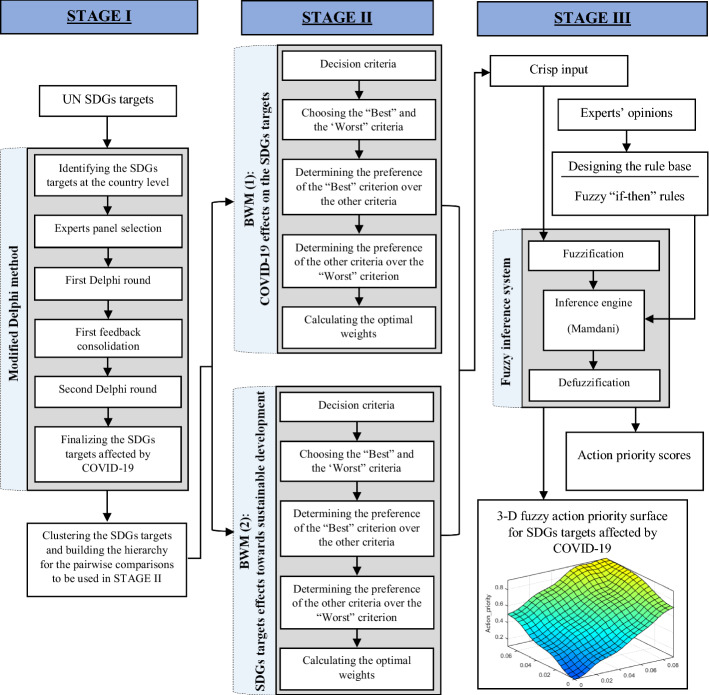
Research framework

The research framework we developed for our study adopts a mixed-method approach that consists of three stages, as shown in Fig. 1. These are Stage I: Using a modified Delphi survey to identify the SDGs targets whose achievement has been affected by COVID-19; Stage II: Using best–worst method (BWM) to calculate the weights of the COVID-19 effects on the achievement of the identified SDGs targets, and also the weights of the impact of these SDGs targets on the sustainable development; and finally, Stage III: Using a fuzzy inference system (FIS) to plot the identified SDGs targets on a three-dimensional (3-D) fuzzy action priority surface. The methods applied in these stages are described in the following subsections.

### Modified Delphi survey

The Delphi method is a scientific process to collect, manage, and analyze opinions from expert panels (Ahmad & Wong, 2019; Esfandabadi & Esfahani, 2018) in an interactive but anonymous multistage forecasting structure (Fritschy & Spinler, 2019). For the first stage of our study, the modified Delphi survey helps to effectively identify the main targets associated with the UN SDGs, whose achievement toward sustainable development has been impacted by the restrictions imposed by the COVID-19 crisis. The term “modified” in the Delphi we used compared to the classic Delphi refers to the use of a pre-generated list of the items in the first round (Varndell et al., 2020), which includes SDGs targets whose impact focus at the country level.

The Delphi survey in our research is conducted in two rounds to reach a consensus among the expert panel. In the first round, the questionnaires are sent to the experts and their responses are gathered. In the second round, the statistical results of the first round survey are given to the experts to give them the chance of rethinking and therefore making it potentially easier to reach a consensus (Chen et al., 2020; Esfandabadi et al., 2020) on the SDGs targets selection. The six steps of the modified Delphi survey conducted in this research are as follows:

- *Step 1*. Identifying the UN SDGs targets whose outcome focus at the country level.
- *Step 2*. Selection of the expert panel in the three main areas of sustainable development.
- *Step 3*. First Delphi round: send the online questionnaire to the experts.
- *Step 4*. Consolidation and integrating the experts’ responses.
- *Step 5*. Second Delphi round: send the integrated feedback and statistical results of the first Delphi round back to the first round experts to make potential changes.
- *Step 6*. Finalize the list of the verified affected SDGs targets by the experts.

The outcome of the modified Delphi method at this stage is the list of SDGs targets whose achievement has been challenged by COVID-19 implications. These targets are then clustered to make the pairwise comparisons within the next stage BWM models easier for the expert panel.

### Best–worst method

The recently developed BWM by Rezaei (2015) to solve multi-criteria decision-making (MCDM) problems has been used for the second stage of our study. The BWM has been widely utilized by scholars for MCDM in different areas of sustainability research, including sustainability assessment (Ren et al., 2017), sustainable supplier selection (Ecer & Pamucar, 2020), sustainable manufacturing (Malek & Desai, 2019), and social sustainability of supply chains (Badri Ahmadi et al., 2017). In comparison with other pairwise comparison-based MCDM methods such as analytic hierarchy process (AHP), BWM (1) performs remarkably better, which becomes obvious when looking at the evaluation criteria, such as consistency ratio, total deviation, minimum violation, and conformity; (2) requires less pairwise comparison data compared to a full pairwise comparison matrix used by AHP; and (3) produces more reliable results by generating more consistent comparisons (Rezaei, 2015). As we expect to evaluate quite a few UN SDGs targets, BWM was selected among MCDM methods to benefit from the advantage of less pairwise comparisons by experts with more reliable results.

For the second stage of this study, the BWM is applied two times to weight COVID-19 effects on the SDGs targets and to weight the SDGs targets on sustainable development, respectively. The five steps of the BWM (Rezaei, 2015, 2016) applied in our research are presented as follows:

*Step 1*. A set of decision criteria is identified. The set of SDGs targets whose achievement has been affected by COVID-19 has been extracted from the Delphi method output generated in the previous stage as the decision criteria set for this step. To facilitate the pairwise comparisons by the experts, these targets were put into clusters of sub-criteria, and the main cluster themes were considered as the main criteria, forming a hierarchy.

*Step 2*. The best and the worst criteria are chosen. In this step, for each cluster of the first BWM model (weighting COVID-19 effects on the SDGs targets), the most impacted SDGs target by COVID-19 (best) and also the least impacted SDGs target by COVID-19 (worst), and for each cluster of the second BWM model (weighting the SDGs targets on sustainable development), and the most important SDGs target toward sustainable development (best) and the least important one (worst) is identified by the decision-makers without any comparison.

*Step 3*. The preference of the best criterion over all the other decision criteria (SDGs targets) is determined using a number between 1 (which means equal preference) and 9 (which means maximum preference) for each cluster and theme group. The resulting best-to-others vector in this step would be: $A_{B} = \left( {a_{B1} ,a_{B2} , \ldots ,a_{Bn} } \right)$, where $a_{Bj}$ indicates the preference of the best criterion *B* over criterion *j*, and accordingly $a_{BB} = 1$.

*Step 4*. The preference of all the criteria over the worst criterion is determined using a number between 1 and 9 for each of the clusters and the group of main criteria. The resulting others-to-worst vector would be: $A_{W} = \left( {a_{1W} ,a_{2W} , \ldots ,a_{nW} } \right)^{T}$, where $a_{jW}$ indicates the preference of the criterion *j* over the worst criterion *W*, and accordingly $a_{WW} = 1$.

*Step 5.* Finally, the optimal weights $\left( {w_{1}^{*} ,w_{2}^{*} , \ldots ,w_{n}^{*} } \right)$ for the criteria are determined. The ideal solution for each pair of $w_{B} /w_{j}$ and $w_{j} /w_{w}$ would be where $w_{B} /w_{j} = a_{Bj}$ and $w_{j} /w_{w} = a_{jw}$. Therefore, the maximum among the set of $\left\{ {\left| {w_{B} - a_{Bj} w_{j} } \right|, \left| {w_{j} - a_{jw} w_{w} } \right|} \right\}$ should be minimized, and the following formulation can be considered for the problem (Eq. (1)):

1 $$\begin{aligned} & \min {\text{max}}_{j} \left\{ {\left| {w_{B} - a_{Bj} w_{j} } \right|,\left| {w_{j} - a_{jw} w_{w} } \right|} \right\} \\ & {\text{s.t.}} \\ & \mathop \sum \limits_{j} w_{j} = 1 \\ & w_{j} \ge 0,\quad {\text{for}}\;{\text{all}}\;j \\ \end{aligned}$$

This problem can then be translated into a linear programming problem as Eq. (2):

2 $$\begin{aligned} & \min \xi^{L} \\ & {\text{s.t.}} \\ & \left| {w_{B} - a_{Bj} w_{j} } \right| \le \xi^{L} ,\quad {\text{for}}\;{\text{all}}\;j \\ & \left| {w_{j} - a_{jw} w_{w} } \right| \le \xi^{L} ,\quad {\text{for}}\;{\text{all}}\;j \\ & \mathop \sum \limits_{j} w_{j} = 1 \\ & w_{j} \ge 0,\quad {\text{for}}\;{\text{all}}\;j \\ \end{aligned}$$

The optimal weights $\left( {w_{1}^{*} ,w_{2}^{*} , \ldots ,w_{n}^{*} } \right)$ and the consistency indicator $\xi^{{L^{*} }}$ would be obtained by solving Eq. (2). The value $\xi^{{L^{*} }}$ shows the reliability of the comparison, and the nearer it is to zero, the more desirable it is. The computed weights are considered as the local weights for the sub-criteria. Therefore, to compute the global weights of the sub-criteria, the local weight of each sub-criterion is multiplied by the weight of its corresponding main criteria.

### Fuzzy inference system

The weights obtained from the two BWM models in the previous stage provide a two-dimensional matrix consisting of COVID-19 effects on the SDGs targets and the SDGs targets achievement effects on the implementation of sustainable development. This matrix can be helpful for the decision-makers to prioritize their managerial actions to focus on each target. However, dividing the matrix into different zones with crisp borders is not sufficiently reliable for classification when experts’ opinion denotes a considerable range (Guðlaugsson et al., 2020).

**Fig. 2:**
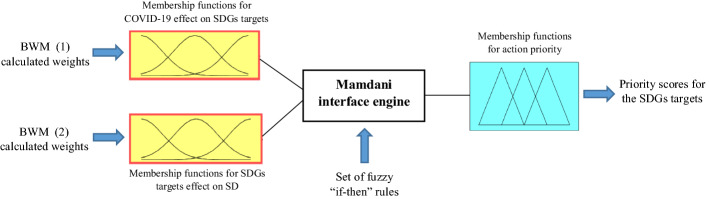
Designed FIS for ranking the priorities of action for the SDGs targets

To overcome this issue, based on the fuzzy logic, a FIS is designed to map the pairs of calculated weights for each SDG target in the BWM models on a 3-D fuzzy action priority surface and obtain its priority score. As illustrated in Fig. 2, the designed FIS in our study works based on the following three main steps: (1) fuzzification of the received crisp BWM weight values as the input, (2) processing the inputs through Mamdani’s rule-base inference engine (Mamdani & Assilian, 1975) that is supplied by rules based on the experts' opinion, and (3) defuzzification of the results and presenting a crisp value as the priority score of each SDG target. Since Gaussian distribution is a reliable option for representing the distributions and uncertainties in real-world problems (Tan et al., 2017), Gaussian membership functions are used for the input and output variables to represent fuzzy sets in the designed FIS.

The final calculated crisp values as the priority scores of the SDGs targets are plotted in a 3-D fuzzy action priority surface, which is explained in detail in Sect. 4.4.

## Results and discussion

To clearly report the results to address the research questions, the results are presented and analyzed in four sections, including expert panel (Sect. 4.1) to answer RQ1, BWM for weighting the SDGs targets affected by COVID-19 (Sect. 4.2) to answer RQ2, the SDGs targets and sustainable development (Sect. 4.3) to answer RQ3, and finally, post-COVID-19 action priority toward sustainable development (Sect. 4.4) to answer RQ4.

### Expert panel

The expert panel of our study consisted of 19 experts from academia, industry, research centers, media, and government sectors in Iran. The experts were identified and invited to participate in the research based on their main field of activity, qualification, and work experience. The responses were gathered using questionnaires from groups of experts in the four rounds of the survey in our research including Delphi round 1 (R1), Delphi round 2 (R2), first BWM, and second BWM from June 27, 2020, to July 14, 2020. Table 1 shows the characteristics of the expert panel and their contribution to our research.

**Table 1:** Expert panel

Expert no.	Field of activity	Affiliation	Qualification	Work experience (years)	Delphi R1 participation	Delphi R2 participation	BWM (1) participants	BWM (2) participation
1	Economy	Academia	PhD	16			✓	✓
2	Economy	Academia	PhD	4	✓	✓	✓	✓
3	Economy	Academia	PhD	3			✓	
4	Economy	Media	MSc	5	✓			✓
5	Economy	Research center	PhD	6			✓	✓
6	Economy	Research center	MSc	13	✓	✓	✓	✓
7	Energy sector	Government sector	BSc	18	✓	✓	✓	✓
8	Energy sector	Government sector	PhD	8	✓	✓	✓	
9	Energy sector	Industry	BSc	20	✓	✓		✓
10	Environment	Academia	PhD	9	✓	✓	✓	
11	Environment	Industry	MSc	8	✓	✓	✓	✓
12	Environment	Research center	Ph.D	2	✓		✓	✓
13	Environment	Research center	MSc	13				✓
14	Health sector	Academia	PhD	7	✓	✓	✓	✓
15	Health sector	Research center	PhD	8			✓	✓
16	Social studies	Academia	PhD	12	✓		✓	✓
17	Social studies	Academia	PhD	6			✓	✓
18	Social studies	Government sector	M.Sc	9			✓	✓
19	Social studies	Media	MSc	14	✓	✓	✓	✓
		Total number of participants			12	9	16	16

#### Delphi results

The focus of our research is on the SDGs targets whose outcome focus at the country level in Iran. Therefore, as the first filter for target selection, before using the modified Delphi method, we just considered 94 out of 169 SDGs targets which work at the country level based on the research conducted by McArthur and Rasmussen (2019). The list of these 94 SDGs targets was used to build a questionnaire for the Delphi survey to find the targets, whose achievements have been affected by COVID-19. During two rounds of Delphi, involving 12 experts in the first and 9 in the second round (Table 1), the final list of 26 SDGs targets affected by COVID-19 was identified. The selected 26 SDGs targets and their description based on the UN’s 2030 Agenda for Sustainable Development are presented in “Appendix A.”

**Fig. 3:**
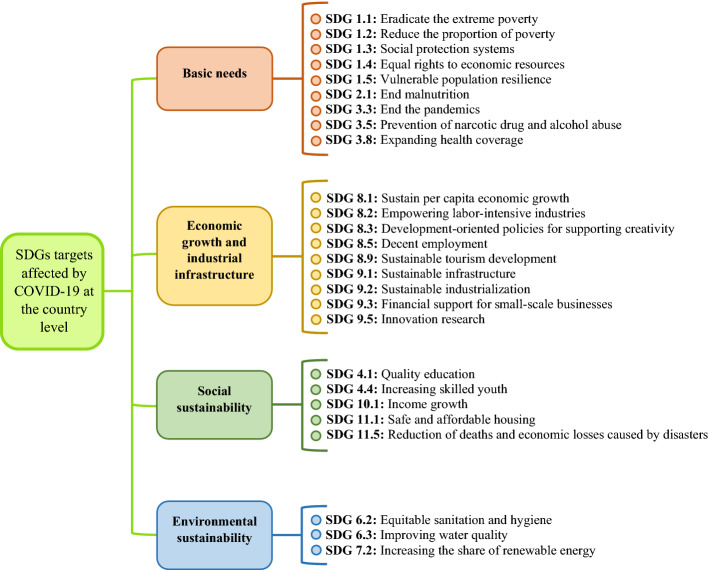
The hierarchical tree for the identified SDGs targets affected by COVID-19

Adopting the SDG clusters proposed by Kostoska and Kocarev (2019) and the UN General Assembly (2015), the 26 identified SDGs targets were assigned by a group of two experts to four clusters including *basic needs*, *economic growth and industrial infrastructure*, *social sustainability*, and *environmental sustainability*. Therefore, the hierarchy of the SDGs targets affected by COVID-19 at the country level was built (Fig. 3), including the four clusters as main criteria, each of which including some sub-criteria (SDGs targets) to be used in the BWM models in the next stage of our research.

### BWM for weighting the SDGs targets affected by COVID-19

Following the BWM steps explained in the methodology section, the first BWM model was solved based on the data gathered from the participating experts (Table 1) to weight the SDGs targets affected by COVID-19 in Iran. The calculated weights of the four main criteria are presented in Table 2.

**Table 2:** Weights of the main criteria for the first BWM model

	Basic needs	Economic growth and industrial infrastructure	Social sustainability	Environmental sustainability	$\xi^{L*}$
Mean	0.357	0.409	0.181	0.054	0.087
Median	0.302	0.440	0.152	0.050	0.084
Standard deviation	0.110	0.100	0.052	0.010	0.018

The obtained mean values show that *economic growth and industrial infrastructure* is the most affected main criterion of SDGs targets followed by *basic needs*. The third and fourth positions refer to *social sustainability* and *environmental sustainability*, respectively, with a considerable distance from the first- and second-ranked items. However, *environmental sustainability*, as the least affected main criterion, has also the lowest value for the standard deviation, which conveys that the expert’s opinions are very close to the mean. Moreover, the $\xi^{{L{*}}}$ values indicate the consistency of the comparisons made by the experts. Since the $\xi^{{L{*}}}$ values for the comparisons made between pairs of the main criteria reported in Table 2 are very close to zero, high consistency of the comparisons can be inferred, which makes the results reliable (Rezaei, 2015, 2016).

In addition to the weights for the main criteria, the local weights for the sub-criteria were also calculated. The mean, median, and standard deviation values of $\xi^{{L{*}}}$ for each of the clusters of sub-criteria are presented in Table 3, indicating the reliability of the results.

**Table 3:** Consistency (${\upxi }^{{\text{L*}}}$) of the clusters of SDGs targets for the first BWM model

	Basic needs cluster	Economic growth and industrial infrastructure cluster	Social sustainability cluster	Environmental sustainability cluster
Mean	0.055	0.051	0.084	0.085
Median	0.051	0.050	0.084	0.082
Standard deviation	0.013	0.004	0.019	0.030

**Fig. 4:**
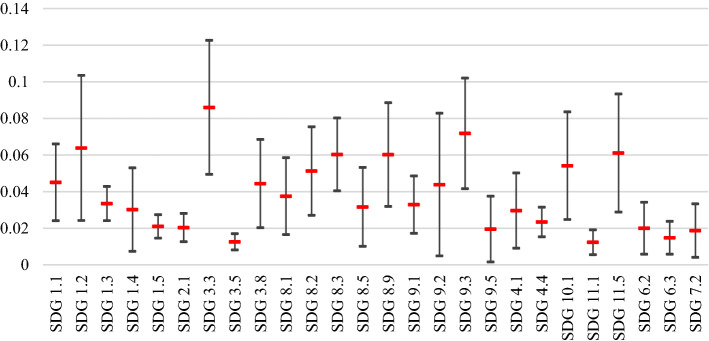
Visualization of the global weights of the sub-criteria (mean and standard deviation) for the first BWM model

After computing the weights for the main criteria and also the local weights for the sub-criteria, the global weights of the sub-criteria were calculated as the multiplication of each sub-criteria local weight and the weight of its corresponding main criteria. Table 4 presents the mean, median, and standard deviation of the global weights of the sub-criteria, and Fig. 4 visualizes the standard deviation of the calculated weights.

**Table 4:** Global weights of the sub-criteria for the first BWM model

Sub-criteria	Mean	Median	Standard deviation
SDG 3.3	0.086	0.076	0.037
SDG 9.3	0.072	0.064	0.030
SDG 1.2	0.064	0.048	0.040
SDG 11.5	0.061	0.057	0.032
SDG 8.3	0.060	0.060	0.020
SDG 8.9	0.060	0.057	0.028
SDG 10.1	0.054	0.056	0.029
SDG 8.2	0.051	0.046	0.024
SDG 1.1	0.045	0.045	0.021
SDG 3.8	0.044	0.041	0.024
SDG 9.2	0.044	0.027	0.039
SDG 8.1	0.038	0.028	0.021
SDG 9.1	0.033	0.031	0.016
SDG 1.3	0.033	0.030	0.009
SDG 8.5	0.032	0.026	0.022
SDG 4.1	0.030	0.025	0.021
SDG 1.4	0.030	0.022	0.023
SDG 4.4	0.023	0.020	0.008
SDG 1.5	0.021	0.017	0.006
SDG 2.1	0.020	0.017	0.008
SDG 9.5	0.020	0.012	0.018
SDG 6.2	0.020	0.012	0.014
SDG 7.2	0.019	0.014	0.015
SDG 6.3	0.015	0.013	0.009
SDG 3.5	0.013	0.011	0.004
SDG 11.1	0.012	0.011	0.007

Based on the obtained global weights (Table 4), SDG 3.3 is the most affected SDG target by COVID-19, followed by SDG 9.3, and SDG 1.2. Moreover, the least affected SDGs targets by COVID-19 are SDG 11.1, followed by SDG 3.5, and SDG 6.3.

In the basic needs cluster, SDG 3.3, which refers to *ending the epidemic and pandemic of any communicable diseases for societies* has been determined as the most impacted SDG target by COVID-19 within the 2030 Agenda for Sustainable Development. On the other side, SDG 3.5 representing *strengthening the prevention of narcotic drug and alcohol abuse* has been identified as the least impacted SDG target by COVID-19.

In the economic growth and industrial infrastructure cluster which is the most affected cluster, SDG 9.3 and SDG 9.5 are the most and the least impacted targets by COVID-19, respectively. SDG 9.3 focuses on the *access of small-scale businesses to financial services and supports*, which has been restricted due to the economic pressure of COVID-19 on the global community and business activities. SDG 9.5, referring to the *encouragement of innovation, research, and development activities in the industrial sector* in Iran, has been identified to be less affected than the other targets in this cluster.

SDG 11.5, as the most impacted target of the social sustainability cluster by COVID-19, points to the *reduction of deaths and economic losses caused by disasters*, while SDG 11.1, which indicates the *access to safe and affordable housing for all people,* is the least impacted one. The number of SDGs targets affected by COVID-19 in the environmental sustainability cluster is less than the other three clusters. This cluster has not been affected considerably by COVID-19 based on the calculated weights for COVID-19 effects on the SDGs targets. The *access to equitable sanitation and hygiene for all people*, representing SDG 6.2, is the most impacted target, while *improving water quality*, representing SDG 6.3, is the least impacted one by COVID-19 in the environmental sustainability cluster.

### SDGs targets and sustainable development

The same process as for the first BWM was used for the second BWM, this time to weight the effects of the 26 identified SDGs targets achievement on the implementation of sustainable development in Iran. Table 5 presents the mean, median, and standard deviation for each of the four main criteria of the pairwise comparison hierarchy and also the consistency of comparisons. As shown in this table, *economic growth and industrial infrastructure* has the highest weight for sustainable development among the four considered criteria, followed by *basic needs* and *social sustainability*. Moreover, *environmental sustainability* has the lowest mean value, and the least standard deviation showing more concentration of the expert’s opinions around the mean.

**Table 5:** Weights of the main criteria for the second BWM model

	Basic needs	Economic growth and industrial infrastructure	Social sustainability	Environmental sustainability	$\xi^{L*}$
Mean	0.343	0.383	0.155	0.119	0.056
Median	0.267	0.466	0.172	0.103	0.052
Standard deviation	0.118	0.127	0.043	0.034	0.012

For each of the clusters of sub-criteria, the values referring to mean, median, and standard deviation of the consistency ($\xi^{{L{*}}}$) are reported in Table 6. These low values of $\xi^{{L{*}}}$ (near to zero) indicate the high consistency of the comparisons taken place in each of the four clusters and therefore highlight the reliability of the obtained results (Rezaei, 2015, 2016).

**Table 6:** Consistency (${\upxi }^{{\text{L*}}}$) of the clusters of SDGs targets for the second BWM model

	Basic needs cluster	Economic growth and industrial infrastructure cluster	Social sustainability cluster	Environmental sustainability cluster
Mean	0.056	0.054	0.081	0.055
Median	0.057	0.054	0.084	0.049
Standard deviation	0.008	0.006	0.018	0.016

The local weights computed for each of the sub-criteria (SDGs targets) in each cluster were multiplied by the relevant weight computed for its main criteria, and the global weights as reported in Table 7 were obtained. As can be seen in this table, the highest weights belong to SDG 1.2, SDG 8.3, and SDG 1.1, while the lowest ones refer to SDG 3.5, SDG 4.1, and SDG 11.1, both respectively. Noticeably, SDG 11.1 and SDG 3.5 had been appeared as the least affected targets in the first BWM, as well.

**Table 7:** Global weights of the sub-criteria (SDGs targets) for the second BWM model

Sub-criteria	Mean	Median	Standard deviation
SDG 1.2	0.065	0.070	0.031
SDG 8.3	0.064	0.051	0.053
SDG 1.1	0.062	0.053	0.025
SDG 11.5	0.058	0.046	0.022
SDG 9.3	0.054	0.055	0.031
SDG 9.5	0.051	0.044	0.017
SDG 3.8	0.050	0.037	0.033
SDG 7.2	0.048	0.048	0.023
SDG 8.1	0.047	0.042	0.022
SDG 8.5	0.041	0.040	0.028
SDG 6.2	0.040	0.032	0.024
SDG 8.2	0.037	0.028	0.028
SDG 1.3	0.034	0.033	0.014
SDG 3.3	0.034	0.018	0.040
SDG 10.1	0.033	0.029	0.019
SDG 9.1	0.032	0.022	0.023
SDG 6.3	0.031	0.027	0.022
SDG 1.5	0.030	0.024	0.015
SDG 9.2	0.030	0.016	0.032
SDG 1.4	0.029	0.028	0.019
SDG 2.1	0.028	0.021	0.020
SDG 8.9	0.027	0.022	0.018
SDG 4.4	0.025	0.026	0.012
SDG 11.1	0.024	0.021	0.019
SDG 4.1	0.014	0.009	0.012
SDG 3.5	0.011	0.012	0.005

**Fig. 5:**
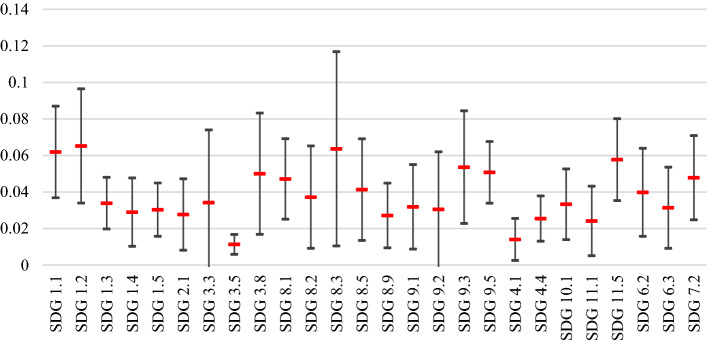
Visualization of the global weights of the sub-criteria (mean and standard deviation) for the second BWM model

Moreover, the visualization of the standard deviation of the calculated weights, as in Fig. 5, shows that the expert’s opinions are much near to each other regarding the SDG 3.5, which is *strengthen the prevention of narcotic drug and alcohol abuse*, while there were many diverse opinions regarding SDG 8.3 representing *development policies for supporting creativity and innovation*.

Finally, the calculated weights of the SDGs targets affected by COVID-19 obtained from the first BWM and also the calculated weights of the SDGs targets achievement toward implementing sustainable development obtained from the second BWM were used to build the FIS in the next stage to prioritize the action toward the SDGs targets within the 2030 Agenda for Sustainable Development considering COVID-19 implications.

### Post-COVID-19 action priority toward sustainable development

At this stage, a FIS was built to weight and rank the priorities of the SDGs targets for action toward the UN’s 2030 Agenda for Sustainable Development. In the development of this FIS, Gaussian membership functions were considered for the effect of COVID-19 on the SDGs targets, the effect of SDGs targets achievement on the implementation of sustainable development, and also for the action priority. Based on the experts’ opinion and considering the results of the two BWM models of the second stage, four membership functions in the range of [0, 0.09] were set for the effect of COVID-19 on the SDGs targets, and three membership functions in the range of [0, 0.07] were set for the effect of the SDGs targets on the achievement of sustainable development. Five Gaussian membership functions were also set for the action priority in the range of [0, 1]. Moreover, to make the Mamdani engine work, 12 “if–then” rules according to the expert’s opinions were fed into the model, linking various levels of input variables with the output, as presented in “Appendix B.”

**Fig. 6.**
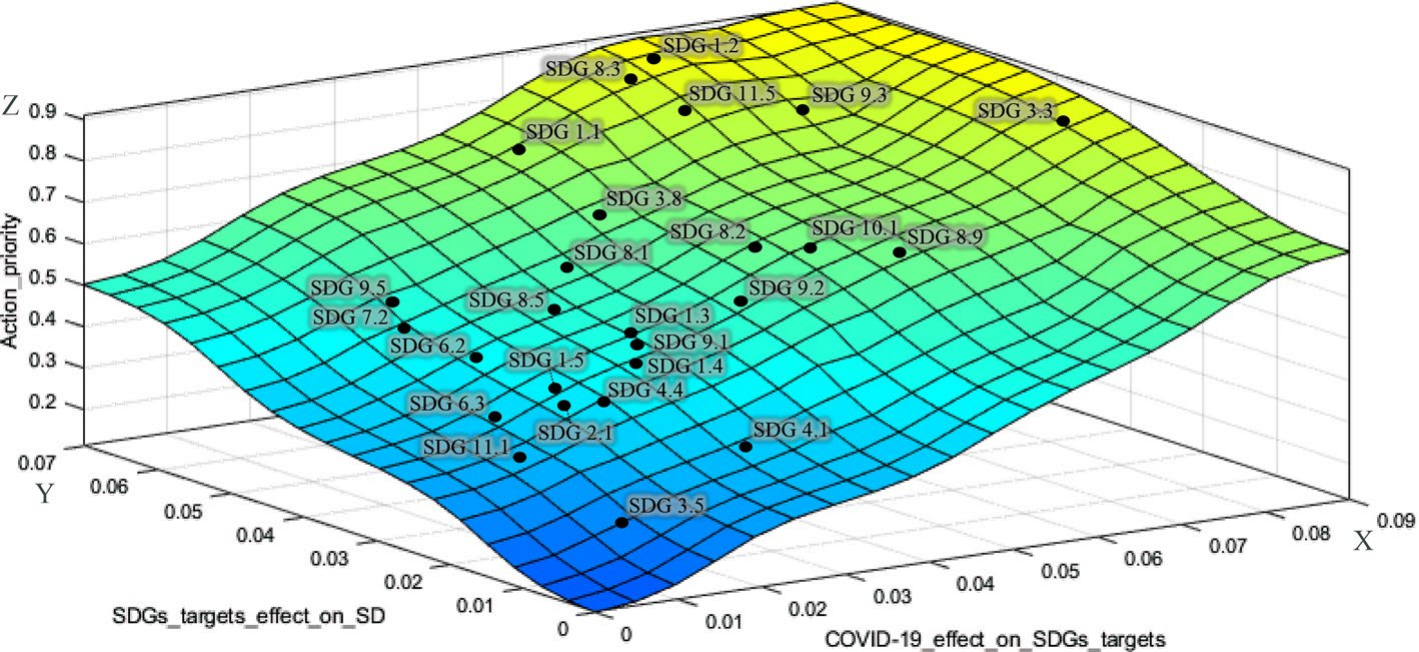
3-D fuzzy action priority surface for the SDGs targets

The surface developed in the designed FIS, as shown in Fig. 6, shows the action priority scores of the SDGs targets (Z-axis) based on the effects of COVID-19 on the SDGs targets (X-axis) and the effects of SDGs targets achievement on the implementation of sustainable development (Y-axis). The results obtained from the two BWM models were plotted on this surface to better illustrate the location of each SDG target on the surface. The surface colors ranging from blue to yellow denote the increase of action priority scores from zero toward one.

**Fig. 7:**
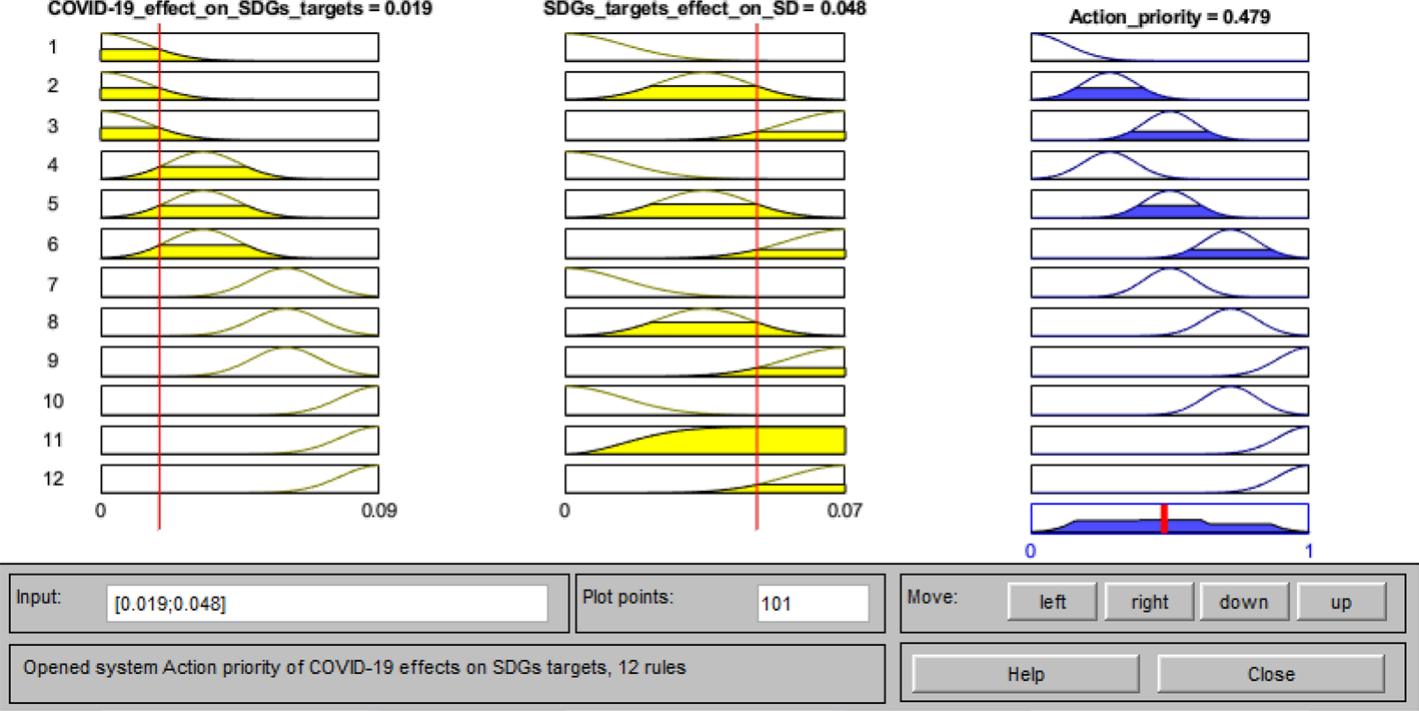
Rule viewer of the FIS

**Fig. 8:**
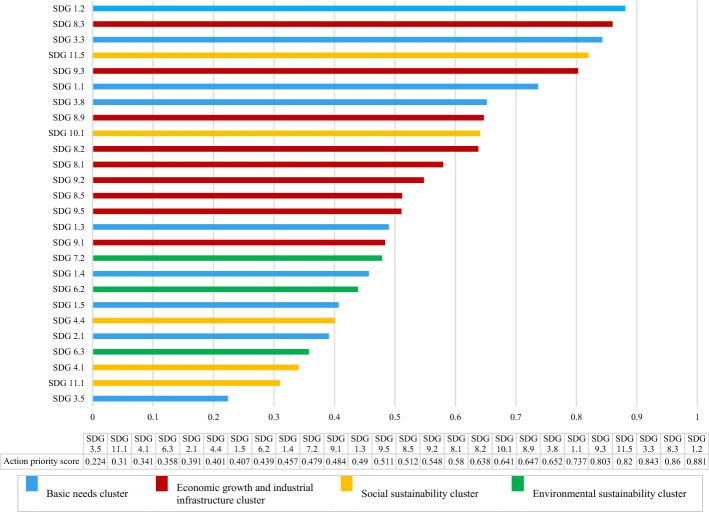
Action priority scores and ranking of the identified SDGs targets affected by COVID-19

To provide crisp values for the action priority scores and rank them accordingly, the pair of values for the SDGs targets obtained from the two BWM models were entered as inputs to the rule viewer of the FIS, as shown in Fig. 7. The system maps the input values on their relevant fuzzy membership functions and provides the crisp value of action priority of the SDGs targets. By following this procedure for all the 26 SDGs targets, their action priority scores were obtained and are ranked as shown in Fig. 8.

In fact, Fig. 8 presents the main output of our research, which is the priority values calculated for the SDGs targets considering the restrictions imposed by the COVID-19 pandemic crisis on the 2030 Agenda for Sustainable Development in Iran. Government, stakeholders, and policy-makers can better decide about the modification of the sustainable development action plan based on the presented guideline and research framework for prioritizing the SDGs targets for action.

The SDGs targets are ranked based on the priority scores in Fig. 8. The first five SDGs targets in this figure including SDG 1.2, SDG 8.3, SDG 3.3, SDG 11.5, and SDG 9.3, which have been also plotted in the yellow area of the fuzzy surface (Fig. 6), are identified as the high priorities for action in the recovery agenda for sustainable development post COVID-19.

The highest priority for action refers to SDG 1.2 from the *basic needs* cluster of the SDGs. The focus of this target is on *reducing the proportion of poor people at least by half* based on the national definition in Iran. SDG 8.3, which belongs to the *economic growth and industrial infrastructure* cluster, is the second priority for action. This target represents *development-oriented policies that support innovation and creativity for productive activities in small- and medium-sized enterprises*. Then, *making efforts to end pandemics, epidemics, and infectious diseases* is the third priority for action toward sustainable development, which is denoted by SDG 3.3 from the *basic needs* cluster. This target has attracted much attention compared with the past due to the serious threats for global health caused recently by COVID-19. SDG 11.5 from the *social sustainability* cluster is ranked as the fourth priority for action. This target concentrates on the *reduction of deaths and economic losses caused by disasters*. COVID-19, as a shocking disaster, has led to serious economic losses for many people especially those who are in a vulnerable situation, which should be considered by the government and policy-makers for providing support. The last high priority for action goes to SDG 9.3 that refers to *increasing the financial services for small-scale industrial enterprises* and belongs to the *economic growth and industrial infrastructure* cluster. During the economic pressure and restrictions caused by COVID-19, small-scale industrial units have been facing many challenges, and the financial support provided by the government is vital for their survival in the industry. Therefore, these SDGs targets (SDG 1.2, SDG 8.3, SDG 3.3, SDG 11.5, and SDG 9.3) should be put in priority for action by all sustainable development stakeholders and policy-makers in the sustainable development recovery agenda post COVID-19.

The SDGs targets that have been plotted in the light green area of the fuzzy surface (Fig. 6) between light blue and yellow are considered as the upper-middle priorities for action including SDG 1.1, SDG 3.8. SDG 8.9, SDG 10.1, SDG 8.2, SDG 8.1, and SDG 9.2. SDG 1.1 and SDG 3.8 belonging to the *basic needs* cluster specifically highlight the efforts for eradication of the extreme poverty in the society and *expanding health coverage* that has been affected by COVID-19. In the *economic growth and industrial infrastructure cluster*, SDG 8.9, SDG 8.2, SDG 8.1, and SDG 9.2 have been identified as upper-middle priorities for action. SDG 8.9 refers to *sustainable tourism development*, which is one of the most impacted areas by COVID-19 due to the loss of many jobs and local businesses. *Empowering labor-intensive industries by technological diversification to achieve higher economic productivity* and *sustain per capita economic growth*, as stressed in SDG 8.2 and SDG 8.1, are the next priorities within this cluster. The last upper-middle priority for action within the *economic growth and industrial infrastructure* cluster goes to SDG 9.2, denoting the importance of *sustainable industrialization to increase the gross domestic product*. The achievement of SDG 10.1 as the only identified target from the *social sustainability* cluster in the upper-middle level of priority, which stands for *faster income growth of the bottom 40 percent of the population compared with the national level*, has become much difficult than ever considering COVID-19 crisis and needs more investment.

The light blue area of the priority surface (Fig. 6), including SDG 8.5, SDG 9.5, SDG 1.3, SDG 9.1, SDG 7.2, SDG 1.4, SDG 6.2, and SDG 1.5, represents the lower-middle priority level for action. *Achieving decent employment for all people* (SDG 8.5), *enhancing innovation by expanding scientific research for industries* (SDG 9.5), and *supporting economic development by developing reliable sustainable infrastructure* (SDG 9.1) from *economic growth and industrial infrastructure cluster* have been identified as the lower-priority for action in comparison with the other SDGs targets in this cluster. SDG 1.3, SDG 1.4, and SDG 1.5 are the lower-priority targets of the *basic needs* cluster, which denote *implementation of social protection systems and equal rights to basic services and economic resources*. According to the results, the *environmental sustainability* cluster has been less affected by COVID-19 than the other clusters. The COVID-19 has not seriously challenged the achievement of SDG 6.2 and SDG 7.2 from this cluster which concentrates on *equitable sanitation and hygiene for all people especially women and girls* and also *increasing the share of renewable energy*. Although Iran is a rich country in terms of renewable energy resources, the required infrastructure for proper deployment of these resources is not well established (Fadai et al., 2011). While the experts think that the environmental pillar has been less affected by COVID-19 in comparison with the economic and social pillars of sustainability, policy-makers should keep caring about the SDGs of the environmental pillar, as well.

The rest of the SDGs targets which have been plotted in the bottom of the fuzzy surface (dark blue area in Fig. 6), including SDG 4.4, SDG 2.1, SDG 6.3, SDG 4.1, SDG 11.1, and SDG 3.5, are considered as the lowest priorities for action, respectively. These targets have been mostly less affected than the other SDGs targets by COVID-19. Among these SDGs targets, SDG 4.1 and SDG 4.4 from the *social sustainability* cluster highlight the *quality education* and *increasing use of the capabilities of young generations with relevant skills*. Even though the effect of COVID-19 in Iran has not been critical in this area, policy-makers and education officials should make effort to support e-learning and online education with a special focus on vulnerable children. Table 8 summarizes the priority levels for action for all 26 studied SDGs targets.

**Table 8:** Summarizing SDGs targets priority levels

Priority level	SDGs targets (sorted from high to low priority)
High	SDG 1.2, SDG 8.3, SDG 3.3, SDG 11.5, SDG 9.3
Upper-middle	SDG 1.1, SDG 3.8, SDG 8.9, SDG 10.1, SDG 8.2, SDG 8.1, SDG 9.2
Lower-middle	SDG 8.5, SDG 9.5, SDG 1.3, SDG 9.1, SDG 7.2, SDG 1.4, SDG 6.2
Low	SDG 1.5, SDG 4.4, SDG 2.1, SDG 6.3, SDG 4.1, SDG 11.1, SDG 3.5

## Conclusion

Since the announcement of COVID-19 as a pandemic by WHO in March 2020, its widespread adverse implications for the global community and business activities have been increasingly paid attention to across the world. The shared blueprint of the 2030 Agenda for Sustainable Development adopted by the UN in 2015 has been significantly affected by the restrictions imposed by COVID-19 on the economy and societies. The 2030 Agenda for Sustainable Development needs to be supported by governments and sustainable development stakeholders for providing significant financial resources. Moreover, the financial burden caused by COVID-19 on the global community has faced the achievement of the SDGs and their associated targets a significant challenge, especially in less-developed and developing countries. In such a situation, effectively prioritizing the actions for sustainable development plays a vital role to overcome these newly emerged financial limitations and support the SDGs achievement.

This research was conducted, as a response to the urgent call for recovery action against the COVID-19 crisis, with the main aim of mapping the effects of COVID-19 on the sustainable development roadmap in Iran focusing on the UN’s SDGs targets whose outcomes appear at the country level. Applying a mixed-method approach, including the Delphi method, BWM, and FIS, a fuzzy action priority surface was built. As a result, the 26 identified SDGs targets (out of the 94 at the country level) affected by COVID-19 were prioritized for action toward implementing the 2030 Agenda for Sustainable Development.

*Economic growth and industrial infrastructure* was identified as the most affected cluster of the SDGs targets by COVID-19 followed by *basic needs*, *social sustainability,* and *environmental sustainability*, respectively. The SDGs targets within the clusters, which were considered as the sub-criteria in the hierarchical tree for the MCDM approach in this research, were weighted according to their importance in the implementation of the 2030 Agenda for Sustainable Development, and also according to the impact of the pandemic crisis on them. Building a fuzzy surface based on the experts’ opinion and mapping the weights of the SDGs targets on the surface, four priority levels for action from high to low were proposed for them. Based on the results, *Reduction of poor people proportion by half* (SDG 1.2), *development-oriented policies for supporting creativity and job creation* (SDG 8.3), *end the pandemics and other epidemics* (SDG 3.3), *reduction of deaths and economic loss caused by disasters* (SDG 11.5), and *financial support for small-scale enterprises* (SDG 9.3) were identified as the highest priorities for action, respectively.

### Policy and managerial implications

This research contributes to the implementation of the 2030 Agenda for Sustainable Development post COVID-19 in Iran by providing a guideline to prioritize the actions that must be taken by the government and the involved stakeholders considering the pandemic implications for society, the economy, and the environment. The results draw a comprehensive map of the COVID-19 impacts on different areas of the sustainable development action plan and support the relative authorities, and practitioners by presenting a post-COVID-19 agenda to effectively prioritize and manage the targets of the SDGs, within the context of developing countries, Iran in particular. Moreover, due to the financial pressure imposed by the pandemic on the organizations and industries contributing to the 2030 Agenda for Sustainable Development, prioritizing the actions has become more critical than ever. The proposed fuzzy action priority surface structure in our research can serve as a policy response at the country level to guide managers and decision-makers to (i) better plan for allocating the financial and non-financial resources to the SDGs targets affected by COVID-19 to alleviate the adverse environmental, economic, and social impacts of the pandemic, and (ii) adopt more effective strategies to support the SDGs achievement within the tough journey toward the implementation of the 2030 Agenda for Sustainable Development in the post-COVID-19 world.

### Limitations and further research

Our research comes with some limitations which deserve to be more investigated in future research. First, although the presented research framework in our study for mapping the COVID-19 effects on the sustainable development pathway and its associated SDGs targets was based on evidence from Iran, as a developing country, it could be used as a pattern to be customized for any other developing or developed country. The same research for the other developing or developed countries using different panels of experts is recommended to compare the results and evaluate the generalizability of our proposed research framework. Second, our research was focused only on the SDGs and their associated targets within the 2030 Agenda for Sustainable Development and did not consider their relevant indicators. Further study is recommended to investigate the effects of COVID-19 on the specific indicators of those SDGs. Third, our research mapped the effects of COVID-19 on the SDGs targets whose outcomes are focused on the country level and excluded the international ones. Additional research is needed to address the targets excluded from our research, such as SDG 17 which reflects the global partnership between countries for all the other 16 SDGs. Third, the main focus of our study was on prioritizing the SDGs targets for action considering COVID-19 effects. Further investigation regarding policy and strategy development based on the obtained action priority in our research is encouraged for future research. And finally, although this research presented a general picture of the COVID-19 implications for the achievement of the SDGs targets, more detailed studies focusing on every single one of the 17 SDGs are deserved to be conducted considering the COVID-19 crisis.

## Code availability:

MATLAB software is used for Fuzzy Inference System (FIS), and the rules applied in FIS are provided in “Appendix B” of the article.

## Appendix A

See Table

**Table 9:** Identified SDGs targets affected by COVID-19 at the country level

SDG	Target no.	Target description based on the UN 2030 Agenda for Sustainable Development^a^
SDG 1	SDG 1.1	“By 2030, eradicate extreme poverty for all people everywhere, currently measured as people living on less than $1.25 a day”
	SDG 1.2	“By 2030, reduce at least by half the proportion of men, women and children of all ages living in poverty in all its dimensions according to national definitions”
	SDG 1.3	“Implement nationally appropriate social protection systems and measures for all, including floors, and by 2030 achieve substantial coverage of the poor and the vulnerable”
	SDG 1.4	“By 2030, ensure that all men and women, in particular the poor and the vulnerable, have equal rights to economic resources, as well as access to basic services, ownership and control over land and other forms of 13 property, inheritance, natural resources, appropriate new technology and financial services, including micro-finance”
	SDG 1.5	“By 2030, build the resilience of the poor and those in vulnerable situations and reduce their exposure and vulnerability to climate-related extreme events and other economic, social and environmental shocks and disasters”
SDG 2	SDG 2.1	“By 2030, end hunger and ensure access by all people, in particular the poor and people in vulnerable situations, including infants, to safe, nutritious and sufficient food all year round”
SDG 3	SDG 3.3	“By 2030, end the epidemics of AIDS, tuberculosis, malaria and neglected tropical diseases and combat hepatitis, water-borne diseases and other communicable diseases”
	SDG 3.5	“Strengthen the prevention and treatment of substance abuse, including narcotic drug abuse and harmful use of alcohol”
	SDG 3.8	“Achieve universal health coverage, including financial risk protection, access to quality essential health-care services and access to safe, effective, quality and affordable essential medicines and vaccines for all”
SDG 4	SDG 4.1	“By 2030, ensure that all girls and boys complete free, equitable and quality primary and secondary education leading to relevant and Goal-4 effective learning outcomes”
	SDG 4.4	“By 2030, substantially increase the number of youth and adults who have relevant skills, including technical and vocational skills, for employment, decent jobs and entrepreneurship”
SDG 6	SDG 6.2	“By 2030, achieve access to adequate and equitable sanitation and hygiene for all and end open defecation, paying special attention to the needs of women and girls and those in vulnerable situations”
	SDG 6.3	“By 2030, improve water quality by reducing pollution, eliminating dumping and minimizing release of hazardous chemicals and materials, halving the proportion of untreated wastewater and substantially increasing recycling and safe reuse globally”
SDG 7	SDG 7.2	“By 2030, increase substantially the share of renewable energy in the global energy mix”
SDG 8	SDG 8.1	“Sustain per capita economic growth in accordance with national circumstances and, in particular, at least 7 per cent gross domestic product growth per annum in the least developed countries”
	SDG 8.2	“Achieve higher levels of economic productivity through diversification, technological upgrading and innovation, including through a focus on high-value added and labor-intensive sectors”
	SDG 8.3	“Promote development-oriented policies that support productive activities, decent job creation, entrepreneurship, creativity and innovation, and encourage the formalization and growth of micro-, small- and medium-sized enterprises, including through access to financial services”
	SDG 8.5	“By 2030, achieve full and productive employment and decent work for all women and men, including for young people and persons with disabilities, and equal pay for work of equal value”
	SDG 8.9	“By 2030, devise and implement policies to promote sustainable tourism that creates jobs and promotes local culture and products”
SDG 9	SDG 9.1	“Develop quality, reliable, sustainable and resilient infrastructure, including regional and trans-border infrastructure, to support economic development and human well-being, with a focus on affordable and equitable access for all”
	SDG 9.2	“Promote inclusive and sustainable industrialization and, by 2030, significantly raise industry’s share of employment and gross domestic product, in line with national circumstances, and double its share in least developed countries”
	SDG 9.3	“Increase the access of small-scale industrial and other enterprises, in particular in developing countries, to financial services, including affordable credit, and their integration into value chains and markets”
	SDG 9.5	“Enhance scientific research, upgrade the technological capabilities of industrial sectors in all countries, in particular developing countries, including, by 2030, encouraging innovation and substantially increasing the number of research and development workers per 1 million people and public and private research and development spending”
SDG 10	SDG 10.1	“By 2030, progressively achieve and sustain income growth of the bottom 40 per cent of the population at a rate higher than the national average”
SDG 11	SDG 11.1	“By 2030, ensure access for all to adequate, safe and affordable housing and basic services and upgrade slums”
	SDG 11.5	“By 2030, significantly reduce the number of deaths and the number of people affected and substantially decrease the direct economic losses relative to global gross domestic product caused by disasters, including water-related disasters, with a focus on protecting the poor and people in vulnerable situations”

^a^The descriptions of the targets are based on General Assemly, 2015. Resolution adopted by the General Assembly on 1 September 2015, General Assebly, United Nations. 10.1017/s0020818300024012

^9^.

## Appendix B

See Table

**Table 10:** Fuzzy rules in the designed FIS

No.	Rule		
1	If (COVID-19-effect_on_SDGs_targets is low)	and (SDGs-targets_effect_on_SD is low),	then (Action_priority is very low)
2	If (COVID-19-effect_on_SDGs_targets is low)	and (SDGs-targets_effect_on_SD is medium),	then (Action_priority is low)
3	If (COVID-19-effect_on_SDGs_targets is low)	and (SDGs-targets_effect_on_SD is high),	then (Action_priority is medium)
4	If (COVID-19-effect_on_SDGs_targets is medium)	and (SDGs-targets_effect_on_SD is low),	then (Action_priority is low)
5	If (COVID-19-effect_on_SDGs_targets is medium)	and (SDGs-targets_effect_on_SD is medium),	then (Action_priority is medium)
6	If (COVID-19-effect_on_SDGs_targets is medium)	and (SDGs-targets_effect_on_SD is high),	then (Action_priority is high)
7	If (COVID-19-effect_on_SDGs_targets is high)	and (SDGs-targets_effect_on_SD is low),	then (Action_priority is medium)
8	If (COVID-19-effect_on_SDGs_targets is high)	and (SDGs-targets_effect_on_SD is medium),	then (Action_priority is high)
9	If (COVID-19-effect_on_SDGs_targets is high)	and (SDGs-targets_effect_on_SD is high),	then (Action_priority is very high)
10	If (COVID-19-effect_on_SDGs_targets is very high)	and (SDGs-targets_effect_on_SD is low),	then (Action_priority is high)
11	If (COVID-19-effect_on_SDGs_targets is very high)	and (SDGs-targets_effect_on_SD is medium),	then (Action_priority is very high)
12	If (COVID-19-effect_on_SDGs_targets is very high)	and (SDGs-targets_effect_on_SD is high),	then (Action_priority is very high)

10.

## References

[CR1] Ahmad S, Wong KY (2019). Development of weighted triple-bottom line sustainability indicators for the Malaysian food manufacturing industry using the Delphi method. Journal of Cleaner Production.

[CR2] Alibegovic, M., Cavalli, L., Lizzi, G., Romani, I., & Vergalli, S., (2020). COVID-19 & SDGs: Does the current pandemic have an impact on the 17 Sustainable Development Goals? A qualitative analysis, FEEM BRIEF. https://www.feem.it/m/publications_pages/brief07-2020.pdf.

[CR3] Ambade B, Sankar TK, Kumar A, Gautam AS, Gautam S (2021). COVID-19 lockdowns reduce the Black carbon and polycyclic aromatic hydrocarbons of the Asian atmosphere: Source apportionment and health hazard evaluation. Environment, Development and Sustainability.

[CR4] Badri Ahmadi H, Kusi-Sarpong S, Rezaei J (2017). Assessing the social sustainability of supply chains using Best Worst Method. Resources, Conservation and Recycling.

[CR5] Barbier EB, Burgess JC (2020). Sustainability and Development after COVID-19. World Development.

[CR6] Bherwani H, Gautam S, Gupta A (2021). Qualitative and quantitative analyses of impact of COVID-19 on sustainable development goals (SDGs) in Indian subcontinent with a focus on air quality. International Journal of Environmental Science and Technology.

[CR7] Bherwani H, Nair M, Musugu K, Gautam S, Gupta A, Kapley A, Kumar R (2020). Valuation of air pollution externalities: Comparative assessment of economic damage and emission reduction under COVID-19 lockdown. Air Quality, Atmosphere and Health.

[CR8] Chakraborty I, Maity P (2020). COVID-19 outbreak: Migration, effects on society, global environment and prevention. Science of the Total Environment.

[CR9] Changotra R, Rajput H, Rajput P, Gautam S, Arora AS (2020). Largest democracy in the world crippled by COVID-19: Current perspective and experience from India. Environment, Development and Sustainability.

[CR10] Chen K, Ren Z, Mu S, Sun TQ, Mu R (2020). Integrating the Delphi survey into scenario planning for China’s renewable energy development strategy towards 2030. Technological Forecasting and Social Change.

[CR11] Ecer F, Pamucar D (2020). Sustainable supplier selection: A novel integrated fuzzy best worst method (F-BWM) and fuzzy CoCoSo with Bonferroni (CoCoSo’B) multi-criteria model. Journal of Cleaner Production.

[CR36] Esfandabadi ZS, Esfahani MMS (2018). Identifying and classifying the factors affecting risk in automobile hull insurance in Iran using fuzzy Delphi method and factor analysis. Journal of Industrial Engineering and Management.

[CR12] Esfandabadi ZS, Ranjbari M, Scagnelli SD (2020). Prioritizing risk-level factors in comprehensive automobile insurance management: A hybrid multi-criteria decision-making Model. Global Business Review.

[CR13] Fadai D, Esfandabadi ZS, Abbasi A (2011). Analyzing the causes of non-development of renewable energy-related industries in Iran. Renewable and Sustainable Energy Reviews.

[CR14] Fritschy C, Spinler S (2019). The impact of autonomous trucks on business models in the automotive and logistics industry—A Delphi-based scenario study. Technological Forecasting and Social Change.

[CR15] Gautam S (2020). COVID-19: Air pollution remains low as people stay at home. Air Quality, Atmosphere and Health.

[CR16] Gautam S, Hens L (2020). COVID-19: Impact by and on the environment, health and economy. Environment, Development and Sustainability.

[CR17] Gautam S, Trivedi U (2020). Global implications of bio-aerosol in pandemic. Environment, Development and Sustainability.

[CR18] General Assemly. (2015). Resolution adopted by the General Assembly on 1 September 2015, General Assebly, United Nations.

[CR19] Guðlaugsson B, Fazeli R, Gunnarsdóttir I, Davidsdottir B, Stefansson G (2020). Classification of stakeholders of sustainable energy development in Iceland: Utilizing a power-interest matrix and fuzzy logic theory. Energy for Sustainable Development.

[CR20] Gupta A, Bherwani H, Gautam S, Anjum S, Musugu K, Kumar N, Anshul A, Kumar R (2020). Air pollution aggravating COVID-19 lethality? Exploration in Asian cities using statistical models. Environment, Development and Sustainability.

[CR21] Hosseini SE (2020). An outlook on the global development of renewable and sustainable energy at the time of COVID-19. Energy Research and Social Science.

[CR22] Ike M, Donovan JD, Topple C, Masli EK (2019). The process of selecting and prioritising corporate sustainability issues: Insights for achieving the sustainable development goals. Journal of Cleaner Production.

[CR23] Kostoska O, Kocarev L (2019). A novel ICT framework for sustainable development goals. Sustainability.

[CR24] Kumi E, Yeboah T, Kumi YA (2020). Private sector participation in advancing the Sustainable Development Goals (SDGs) in Ghana: Experiences from the mining and telecommunications sectors. Extractive Industries and Society.

[CR25] Malek J, Desai TN (2019). Prioritization of sustainable manufacturing barriers using Best Worst Method. Journal of Cleaner Production.

[CR26] Mamdani EH, Assilian S (1975). An experiment in linguistic synthesis with a fuzzy logic controller. International Journal of Man-Machine Studies.

[CR27] Mann FD, Krueger RF, Vohs KD (2020). Personal economic anxiety in response to COVID-19. Personality and Individual Differences.

[CR28] McArthur JW, Rasmussen K (2019). Classifying Sustainable Development Goal trajectories: A country-level methodology for identifying which issues and people are getting left behind. World Development.

[CR29] Mogaji E (2020). Impact of COVID-19 on transportation in Lagos, Nigeria. Transportation Research Interdisciplinary Perspectives.

[CR30] Rajput H, Changotra R, Rajput P, Gautam S, Gollakota ARK, Arora AS (2020). A shock like no other: coronavirus rattles commodity markets. Environment, Development and Sustainability.

[CR31] Ranjbari M, Esfandabadi ZS, Zanetti MC, Scagnelli SD, Siebers P-O, Aghbashlo M, Peng W, Quatraro F, Tabatabaei M (2021). Three pillars of sustainability in the wake of COVID-19: A systematic review and future research agenda for sustainable development. Journal of Cleaner Production.

[CR32] Ranjbari M, Morales-Alonso G, Shams Esfandabadi Z, Carrasco-Gallego R (2019). Sustainability and the Sharing Economy: Modelling the Interconnections. Dirección Organización.

[CR33] Ren J, Liang H, Chan FTS (2017). Urban sewage sludge, sustainability, and transition for Eco-City: Multi-criteria sustainability assessment of technologies based on best-worst method. Technological Forecasting and Social Change.

[CR34] Rezaei J (2016). Best-worst multi-criteria decision-making method: Some properties and a linear model. Omega (United Kingdom).

[CR35] Rezaei J (2015). Best-worst multi-criteria decision-making method. Omega (United Kingdom).

[CR37] Siegel KM, Bastos MG (2020). When international sustainability frameworks encounter domestic politics: The sustainable development goals and agri-food governance in South America. World Development.

[CR38] Sigala M (2020). Tourism and COVID-19: Impacts and implications for advancing and resetting industry and research. Journal of Business Research.

[CR39] Tan Y, Shuai C, Jiao L, Shen L (2017). An adaptive neuro-fuzzy inference system (ANFIS) approach for measuring country sustainability performance. Environmental Impact Assessment Review.

[CR40] UN. (2018). The sustainable development goals report 2018, United Nations, New York. 10.11260/kenkokyoiku.19.77

[CR41] Van der Waal JWH, Thijssens T (2020). Corporate involvement in sustainable development goals: Exploring the territory. Journal of Cleaner Production.

[CR42] Varndell W, Fry M, Lutze M, Elliott D (2020). Use of the Delphi method to generate guidance in emergency nursing practice: A systematic review. International Emergency Nursing.

[CR43] WHO. (2021a). WHO Coronavirus (COVID-19) Dashboard | WHO Coronavirus Disease (COVID-19) Dashboard [WWW Document]. https://covid19.who.int/ (Retrieved 14 Mar 21).

[CR44] WHO. (2021b). Iran (Islamic Republic of): WHO Coronavirus Disease (COVID-19) Dashboard | WHO Coronavirus Disease (COVID-19) Dashboard [WWW Document]. https://covid19.who.int/region/emro/country/ir (Retrieved 14 Mar 21).

[CR45] World-Bank-Group. (2020). Iran Economic Monitor: Mitigation and Adaptation to Sanctions and the Pandemic [WWW Document]. http://documents.worldbank.org/curated/en/676781543436287317/pdf/Iran-Economic-Monitor-Weathering-Economic-Challenges.pdf

[CR46] Yoshino N, Taghizadeh-Hesary F, Otsuka M (2020). Covid-19 and optimal portfolio selection for investment in sustainable development goals. Finance Research Letters.

